# Observations of movement dynamics of flying insects using high resolution lidar

**DOI:** 10.1038/srep29083

**Published:** 2016-07-04

**Authors:** Carsten Kirkeby, Maren Wellenreuther, Mikkel Brydegaard

**Affiliations:** 1 National Veterinary Institute (DTU VET), Technical University of Denmark, Bülowsvej 27, DK-1870 Frederiksberg C., Denmark; 2 FaunaPhotonics, Ole Maaloes Vej 3, DK-2200 Copenhagen N., Denmark; 3 Section for Evolutionary Ecology and Center for Animal Movement Research (CAnMove), Department of Biology, Lund University, Sölvegatan 37, 22363 Lund, Sweden.; 4 The New Zealand Institute for Plant & Food Research Ltd, 300 Wakefield Quay Port Nelson, Nelson 7010, New Zealand; 5 Lund Laser Centre (LLC), Department of Physics, Lund University, Sölvegatan 14, 22363 Lund, Sweden

## Abstract

Insects are fundamental to ecosystem functioning and biodiversity, yet the study of insect movement, dispersal and activity patterns remains a challenge. Here we present results from a novel high resolution laser-radar (lidar) system for quantifying flying insect abundance recorded during one summer night in Sweden. We compare lidar recordings with data from a light trap deployed alongside the lidar. A total of 22808 insect were recorded, and the relative temporal quantities measured matched the quantities recorded with the light trap within a radius of 5 m. Lidar records showed that small insects (wing size <2.5 mm^2^ in cross-section) moved across the field and clustered near the light trap around 22:00 local time, while larger insects (wing size >2.5 mm^2^ in cross-section) were most abundant near the lidar beam before 22:00 and then moved towards the light trap between 22:00 and 23:30. We could distinguish three insect clusters based on morphology and found that two contained insects predominantly recorded above the field in the evening, whereas the third was formed by insects near the forest at around 21:30. Together our results demonstrate the capability of lidar for distinguishing different types of insect during flight and quantifying their movements.

Insects are integral parts of many ecosystems, both in terms of taxonomic diversity and ecological function^1^. As such, insects directly and indirectly affect many species through predator-prey interactions and as invasive species, disease vectors, pests, pollinators and pathogens^2^. An urgent issue for insect biodiversity conservation and ecosystem management is the rapid distributional change seen in many insect species worldwide, and efforts are underway to predict short- and long-term consequences^2^. One major factor affecting the movement dynamics of insects are increasing global temperatures, which have been documented to cause insect range expansions and shifts in diverse taxa worldwide e.g. ref. 3. Understanding the distributional changes and modifications in abundance patterns are critical for many insect groups For example insects that act as pollinators; the global economic value of insect pollination in 2005 amounted to US$153 billion^4^. It is also important to study insects that cause and carry zoonotic diseases; for instance malaria costs Africa more than US$12 billion every year in lost GDP^5^. Agricultural pests are likewise economically important; for instance the annual control of the diamondback moth *Plutella xylostella* costs approximately US$4–5 billion^6^.

Monitoring movement changes of flying insects and their ecosystem diversity remains a pertinent challenge, however. This is because their often small size, rapid flight behaviour and sometimes nocturnal activity patterns makes it difficult to reliably detect flying insects with the accuracy needed to make firm observations and classifications. Only few studies have until now been able to record the spatially and temporally resolved movement of small-sized insects individually, without relying on indirect observations (e.g. light traps) or disturbing the environment and the insects themselves^7^. Noteworthy previous efforts include the application of radar entomology to study the movement behaviour of insects^8^,^9^,^10^,^11^. These radar studies range from the monitoring of large-scale insect migrations with Doppler weather radars^12^ and the tracking of individual insects tagged with electrical diodes using harmonic radar^13^. Despite these technical advances in remote sensing in the last decades, most current observations of small-sized insects in the field are still largely based on trapping techniques that can both be costly and do not allow for real-time monitoring^7^.

Recent progress in the field of insect movement dynamics has come from new technological approaches and systems that apply remote *light detection and ranging* (lidar)^14^,^15^,^16^,^17^,^18^ appear particularly promising. Such lidar methods can potentially detect multiple individuals simultaneously within a sampling volume of order 1 m^3^. A particularly promising feature of lidar is that it can estimate the Optical Cross Section (OCS) of flying insects directly, thus allowing for identification of insect groups and quantifying their behaviour in the air over time (e.g. movements towards foraging or mating locations). With radar, behavioural assessments can also be achieved, for instance, the insect track and heading direction of unmarked insects can be measured using beam wobbling or polarization^19^. Increased specificity can be achieved with vertical-beam radars of the type that provide information about the targets’ sizes, shapes, and wing-beat frequencies^20^, but observations in the optical region potentially provide richer information owing to the inherent modulation of the radar cross sections (RCS) and wing beat frequency. However, the current understanding is that the RCS does not oscillate due to change of projected wing area but because of muscle contractions changing the water content. Therefore, the details in the RCS modulation signature is not as rich in information compared to the measurement accuracy that can be achieved using the optical region^21^. In addition, the radar probe volumes are also known to be divergent and to have side lobes, therefore, radar entomology studies have primarily targeted migrating insects at higher altitudes and that are thus moving away from any ground clutter.

Like radar, lidar can also be used to estimate the insect wing beat frequency^22^ allowing to distinguish between insects^21^. Recent improvements allowing fast lidar sampling rates in the kHz range further allows to distinguish between species wing and body cross sections^21^ and this also enables the retrieval of wing beat harmonics important for species identification^23^. Thus, lidar techniques can also potentially be used to measure the abundance and spatio-temporal location of insects, both during the day and at night. Despite the promise of this technology, only a few studies have investigated the utility of lidar for insect monitoring so far, e.g. refs 14,19,21,24,25, and no studies have yet compared the accuracy of lidar methods compared to traditional methods. Contrary to radars, lidar beams can be arranged with considerably higher beam control and insect monitoring can be achieved less than a meter over ground^14^. For these reasons, the lidar technology would seem to have particular promise for monitoring small insects (i.e. those too small to carry transponders) that are flying near the ground or vegetation canopy, where normal radar techniques cannot be applied.

Here we apply lidar technology to study insect assemblages in nature and show its utility for quantifying insect activity. In addition, we compare spatially and temporally resolved abundance measures from lidar with that of UV light trap catches. Light traps present one of the traditional direct methods of quantifying flying insects, and have been commonly applied to monitor insects^26^. In our study, we first compare insect abundance over time, and second, analyse the spatio-temporal distribution of insects. Third, we utilise the morphological information from the lidar data and show its utility to form insect groupings based on the wing-beat frequency and the proportions of the body:wing distribution. These groupings are subsequently evaluated in space and time. Lastly, we discuss the utility of lidar as an insect movement and biodiversity tool and highlight some promising future areas that deserve particular attention.

## Materials and Methods

### Lidar and light trap setup

**Figure 1:**
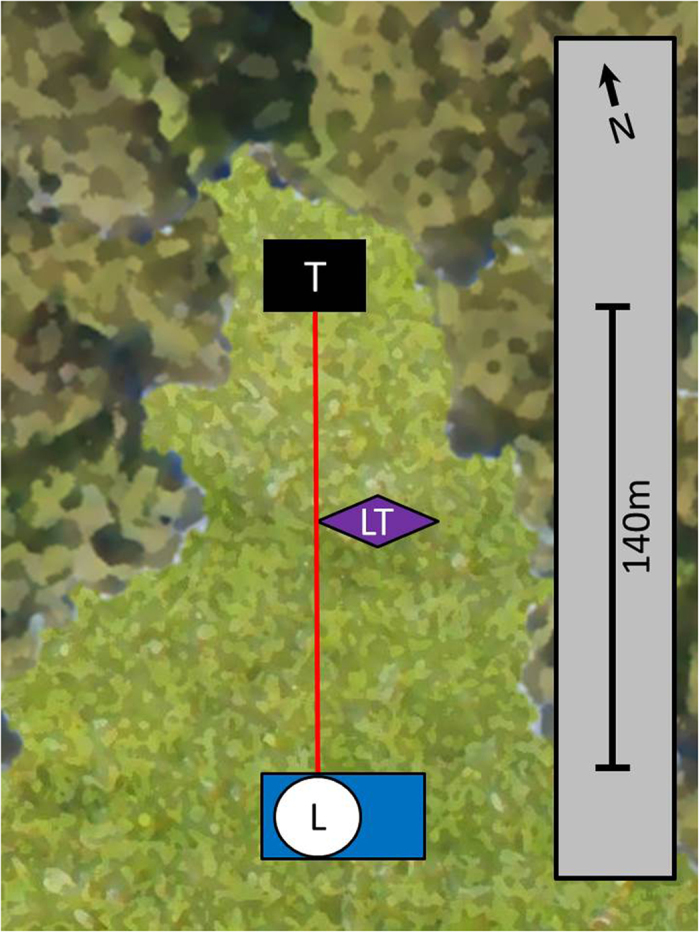
Schematic overview of the Stensoffa study site in southern Sweden. The label L shows the position of the lidar system, T of the termination box, and LT shows the position of the light trap. The red line denotes the area over which the laser beam was directed, and covered a transect that was approximately 140 m long. The dark green area denotes the forest and the light green area the meadow.

Field work was carried out during the night from the 20–21^st^ of July 2013 at the Stensoffa biological field station (N55.6950436, E13.4482755) in southern Sweden. Sweden uses “summertime” during July, so the local time is set one hour forward. Therefore, local time is UTC +2. During the study night, dusk began when the trapping was started at 21:18 and continued until the next trapping period at 22:18 when it was almost dark. The sun set at 21:33 and true midnight was at 01:14. The moon was 95% full and moonrise was at 19:18 and moonset at 03:23. Civil twilight began at 03:58 and ended at 22:25. The lidar system we used is called LUMBO (Lund University Mobile Biosphere Observatory) and part of the Center for Animal Movement Research (CAnMove) at Lund University. The lidar was directed over a meadow, with the beam towards the north, and terminated at 140 m by a box made out of black cardboard. An UV light trap (model Onderstepoort 8 W, 370 (320–420) nm) was positioned 2 m east of the beam at 65 m from the lidar, at a height of 1.8 m and with the direction of the light tube perpendicular to the lidar beam (Fig. 1). The protective and size-selective net on the trap was removed in order to catch all insects approaching the light trap. The light trap was turned on and emptied in batches (see the batch clearing times in Table 1). The time intervals were chosen by continuously evaluating the density of insects in the catches to obtain a balance between obtaining a sufficiently large amount of insects in each batch without disturbing the field too often. Care was taken not to interfere with the beam when emptying the light trap and laser safety goggles were worn at all times.

### Lidar system specifications

The continuous wave (CW) kHz lidar has been described in detail in^19^,^27^,^28^,^29^,^30^. In short, the lidar system was based on ranging by triangulation and imaging the transmitted laser beam on a tilted linear sensor according to the Scheimpflug principle. The transmitter is an 808 nm near infrared 3.2 W CW laser diode expanded to 102 mm diameter. The receiver is a F/5, 102 diameter reflecting telescope (model SkyWatcher, StarTravel), fitted with a RG780 long pass (Schott) filter and an 808 nm, 3 nm full width at half maximum band pass filter (Edmund Optics). The sensor (DALSA Teledyne, Spyder 3, GigE) was a 12-bit line scan camera with 2 × 2048 pixels. Insects can generally perceive light between 300–700 nm^31^ and the majority of insect groups are attracted to light^32^. This is particularly true for flying insects, e.g. moths^33^. In comparison, the lidar beam has a wavelength of 808 nm, compared to 320–420 nm for the light trap. Therefore, the lidar beam itself is not visible to insects, while insects can recognize and navigate towards the light from the trap.

### Light trap insect identification and categorisation

Insect batches were sorted with respect to size into subjectively defined groups for comparison with lidar data and identified according to Chinery^34^. One of the batches (Table 1, batch number 2, caught between 22:18–23:18 local summer time) contained a large number of insects and therefore identification was based on a ¼ subsample. After sorting, the body and wing width and length of one wing was measured on three (where available) randomly chosen specimens from each category and batch, using a Leica MZ5 microscope and the Leica application suite program. The measurements of the widths and lengths were made on wings that were laid flat down (to obtain the widest and longest cross-sectional distances of the wing) and from the lateral side of the body. This was decided because the lidar algorithm estimates the peak sizes of the body and wing OCS. The body and wing measurement were used to estimate the cross-sectional area of the body and wings for each grouping. For insects with two sets of wings, both the fore- and hindwings were included. We used an elliptic approximation to estimate the body and wing cross section area for comparison with the lidar data. We then calculated the body:wing proportions as: *body OCS*/(*body OCS* + *wing OCS*).

### Data analysis

During the lidar experiment, the pixels were binned at 2 × 2 and the line scan camera was operated at a line rate of 5 kHz. The raw data amounted to 340 Gb for the 10 measurement hours. A data reduction and parametrization algorithm was applied following Malmqvist *et al.*^29^. Among other features, this algorithm separates the non-oscillatory body contributions from the oscillatory wing contributions. This is achieved by applying a sliding minimum filter with a width equal to the wing beat period. The processing also includes a calibration to allow the measurement of OCS^25^ and a threshold to separate the insect observations from the static air return.

The backscatter OCS were obtained by terminating the lidar beam at an object with a known Lambertian reflectance. The OCS quantities obtained describe the product resulting from the diffuse reflectance at 808 nm (NIR) and the projected area of the insect. NIR reflectance of insects varies according to their relative melanisation, but on a whole it can be generalised^35^ that it is in the order of 20%. Our configuration of the lidar detects both co- and de-polarized backscatter and thus includes specular reflexes for insects with glossy wings. Specular reflexes can reach 1000% reflectance compared to Lambertian reflectance. Specular reflexes can be distinguished by polarization or higher harmonics, see additional explanations in^21^. Since the OCS relates to the projected sizes and the projection of both the insect wings and body, it becomes larger for dorsal or ventral observations (e.g. we found a large co-variance of these two quantities)^21^. For these reasons, we hypothesized that a unit-less wing-body ratio could be used as a stable measure for separating insect groups. Based on this, we used both a combination of wing-beat frequency and body:wing proportions, and a combination of wing beat frequency and wing area to separate insect groups, as well as the combination of the wing beat frequency and the wing area directly. The signal-to-noise-ratio threshold of >2 is based on temporal statistics and have been described previously^36^,^37^. For each insect observation, a fundamental tone and a discrete harmonic spectrum was fitted^29^.

Data from the light trap counts per time interval were compared with the lidar events. In order to compare insects of the same size between these two methods, the lidar data were filtered to match body morphologies used to categorise insect groupings from the light traps. To recognize the spatio-temporal pattern of different insect groups, the lidar events were divided into two groups: 1) small and fast flapping insects defined by wing beat frequencies above 300 Hz and a cross-sectional wing size below 2.5 mm^2^ and 2), and larger and slow flapping insects defined by wing beat frequencies below 300 Hz and cross-sectional wing size above 2.5 mm^2^. In order to explore the obtained lidar data in further detail, we also took a different approach where the data were clustered based on statistical modes in the wing-beat frequency and body:wing proportions. Clustering was performed by grouping the observations encircled by the three iso-levels.

## Results

A total of 6211 insects were caught with the light trap during the study period. These insects were subsequently separated into 13 groups (see Table 1 for detail). Groups differed to a large extent in their total abundance, with only two specimens caught in the Hemiptera group, whereas 3106 specimens were caught in the “Other Nematocera” group. The time periods at which these different groups were captured also showed large differences (Table 1).

**Figure 2:**
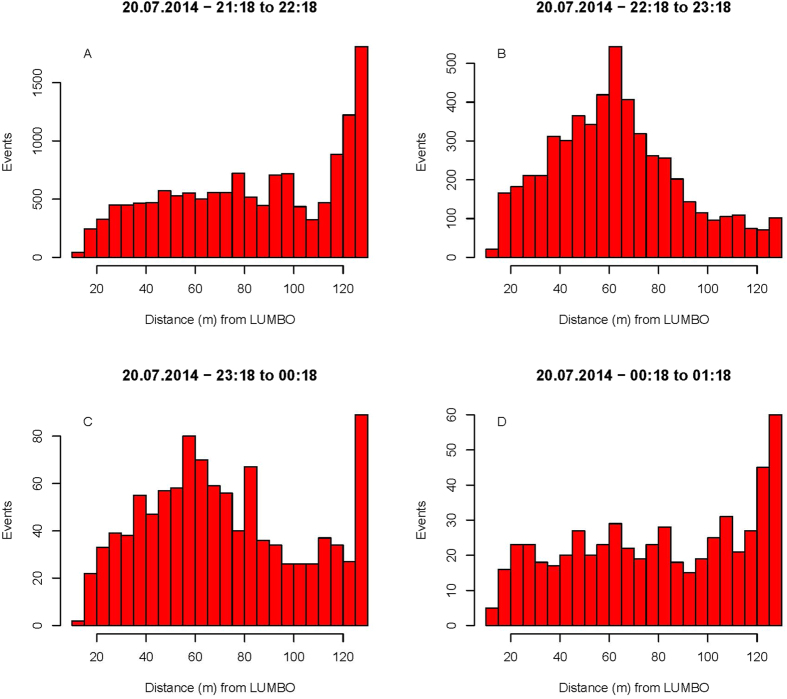
All insect records detected with the lidar between 21:18 and 01:18, in four time periods. LUMBO is the lidar system unit, an acronym for Lund University Mobile Biosphere Observatory. The light trap was placed 65 m from LUMBO. The insect detection peaks from 22:18 to 00:18 show higher insect activity close to the light trap.

**Figure 3:**
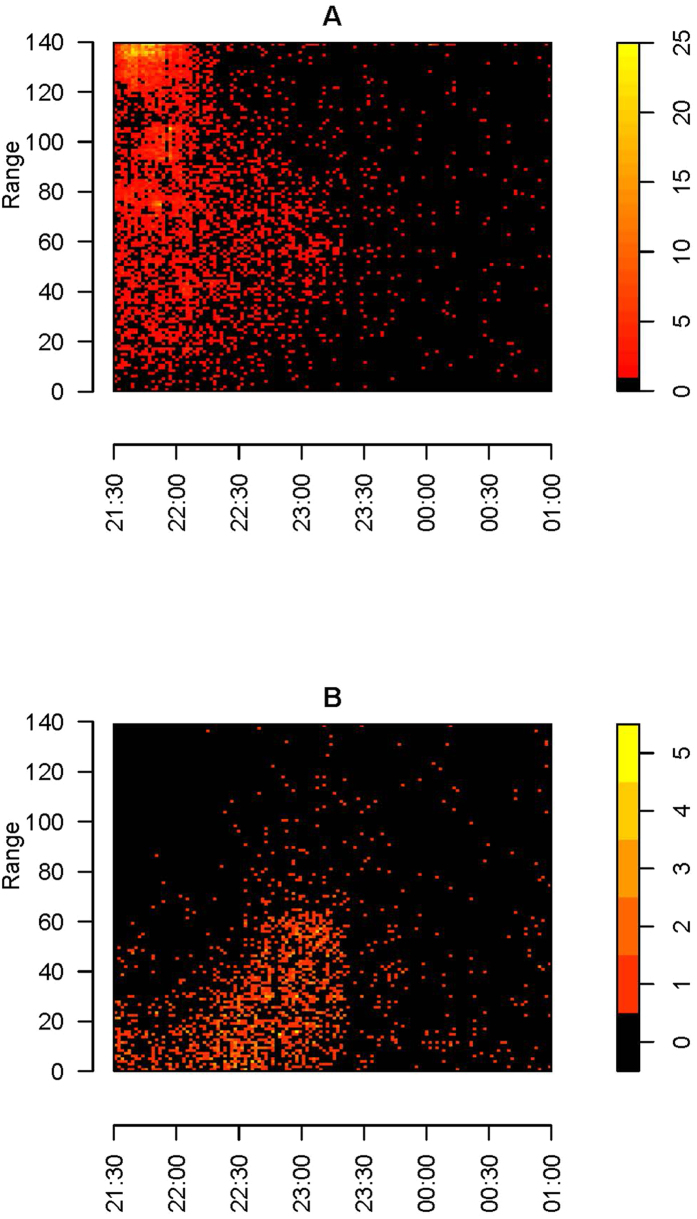
Density plot of (**A**) small insects with a high wing beat frequency >300 Hz and a wing size <2.5 mm^2^ and (**B**) larger insects with a slow wing beat frequency <300 Hz and a wing size >2.5 mm^2^. The x-axis shows the time and the y-axis the spatial range (m) from the lidar system. Sunset was at 21:33 and civil twilight ended at 22:25. The two groupings (**A**,**B**) are also marked in Fig. 6.

A total number of 22808 events (individual insect observations) were recorded with the lidar system, whereof 2052 were recorded within the 5 m range of the light trap position (see Fig. 1 for the spatial setup). Analysis of the lidar data in relations to the spatial distribution within the four time periods of the sampling period is shown in Fig. 2A–D. In Fig. 2A,D, we see that the insect abundance was highest near the termination box in the forest. In Fig. 2B,C a clear peak of insect activity can be seen in the middle of the transect, near the light trap. Furthermore, there was a clear increase in the insect abundance towards the light trap position at about 40 m to each side during these two time periods (Fig. 2). Figure 3 shows the individual events in space and time, where we have divided the recorded events into two groups based on the wing size and wing beat frequency. Two distinct patterns are clear in Fig. 3: A group of small insects (wing size <2.5 mm^2^) with high wingbeat frequency (>300 Hz) were abundant near the trees at a range of 140 m where the beam was terminated. The small sized insect assemblage was abundant near the terminator and near the light trap from dusk to around 22:00. After 22:00 the assemblage was found near the light trap where it disappeared around 23:00. A group of larger insects (wing size >2.5 mm^2^) with slow wing beat frequency (<300 Hz) exhibited quite a different movement behaviour and was most abundant near the lidar during the evening and then moved towards the light trap from around 22:00 onwards until about 23:30 (Fig. 3).

**Figure 4:**
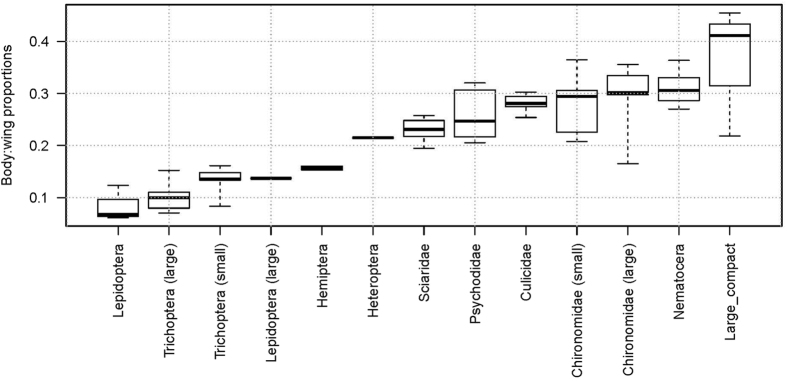
Boxplot of the body:wing proportions of the different insect groups caught in the light trap. Insects were combined from all sampling periods and measured under a dissection microscope.

**Figure 5:**
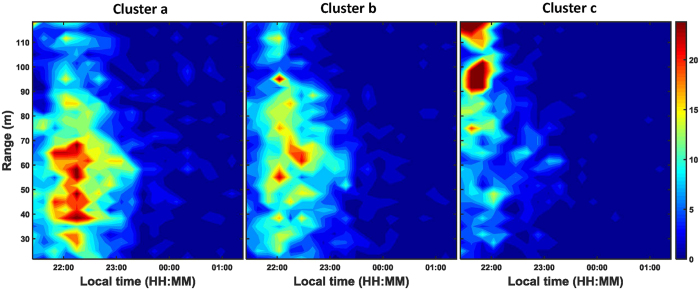
Heatmaps showing cluster a (above), b (middle) and c (bottom) related to the time and spatial range of the insect records. Note the different count scale bars and colour codes, in particular for cluster c. The dotted red line shows the wing beat frequency cut-off used for Fig. 3.

**Figure 6:**
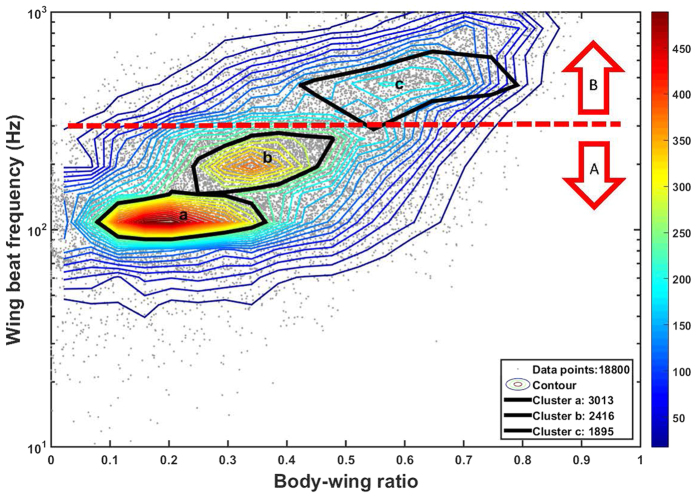
Contour plots showing the point densities found in the lidar data based on the recorded body-wing ratio and the wing beat frequency. Red curves denote areas with a high density of points, whereas blue curves denote a low density points. Black curves indicate the three major insect clusters.

**Figure 7:**
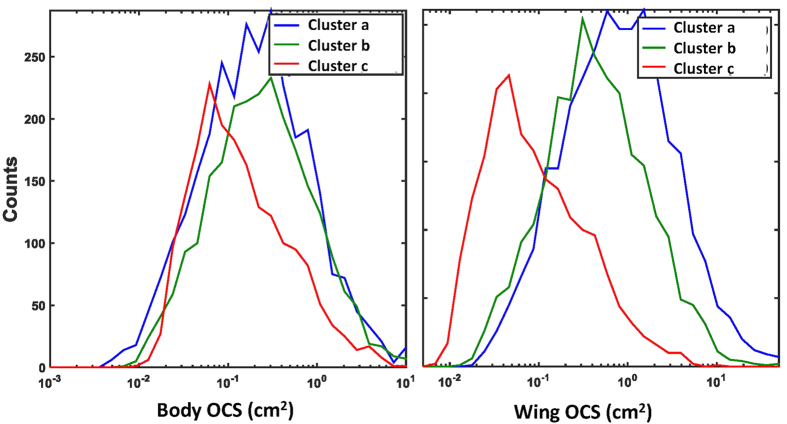
The density of the clusters detected in the lidar data (Fig. 6) shown separately using the body OCS and wing OCS. The absolute optical backscatter cross sections (OCS) values from the body and wing display large overlap between the clusters.

The body:wing proportions based on the morphological measurements are shown in Fig. 4. Unfortunately, all of the morphological groups caught in the light trap have overlapping ranges with other groups and therefore we were not able to use these measurements to match precisely with the lidar dataset. The morphological signal in the lidar data was used to separate the records into three insect non-overlapping clusters based on the spatial resolution of the body:wing proportions and wing beat frequency (Figs 5 and 6). The clusters included 3013, 2416 and 1959 observations and are named cluster a, b and c, respectively. Cluster a consisted of insects with a fundamental (wing beat) frequency of ~120 (80–150) Hz and a body OCS of 30 mm^2^, cluster b consisted of observations with around ~200 (150–300) Hz and a body OCS of 30 mm^2^, and finally cluster c consisted of events with 500 (300–600) Hz frequency and insects with body OCS of 5 mm^2^. We investigated range-time plots for each cluster and found that clusters a and b contained insects that were predominately recorded above the field in the evening, while cluster c contained insects that where abundant near the forest in the beginning of the recordings starting at 21:18 and subsequently moved close to the trap (Fig. 6). The time-range abundance maps for cluster a and b shows increased counts mid-way along the lidar transect (Fig. 5). Such observation could not be explained by system biases or range dependent detection limits of the lidar. Further, we found indications that the spatial width of the distributions changed during the evening. This could either be explained that insects are increasingly attracted to the trap with time or that the trap becomes increasingly efficient as the brightness in the landscape decreased. All three clusters showed a predominant activity pattern before midnight (Fig. 5). Cluster a was most abundant at 30 to 90 m range. Cluster b was most abundant between 50 and 90 m range. Insect activity in cluster c was most abundant near the forest in the beginning of the measuring period at 21.18 PM, just before sunset, and this cluster decreased to a low abundance soon after sunset. We also evaluated body and wing size projections used for clustering separately. As shown in Fig. 7, the clusters found using the body:wing ratio showed substantial overlap.

Based on the body:wing proportions recorded with the lidar for these three clusters, we can estimate which insect groups from the light trap is represented in each cluster. The body:wing proportions of cluster a ranged between 0.10 and 0.35. Based on the median calculated body:wing proportions of the insect groups caught in the light traps (Table 1 and Fig. 4), these clusters thus likely comprise small and large Trichoptera, Aphididae and Heteroptera, large Lepidoptera, *Sciaridae*, *Psychodidae*, *Culicidae*, small and large *Chironomidae* and other Nematocera. Of these, the *Trichoptera* (caddisflies) and *Chironomidae* were by far the most abundant groups in Table 1. The two latter groups have in common that they are nocturnally active, most adults do not feed and they can form swarms^38^. Therefore we assume that the cluster of this group near the forest is related to mating and/or predator-avoidance. The body:wing proportions of cluster b ranges between 0.25 and 0.45 and, hence, likely comprises species from the groups Sciaridae, Psychodidae, Culicidae, Chironomidae, other Nematocera, and other groups characterised by compact insects. The two latter groups were the most abundant groups in this cluster (Table 1) and in Sweden consist of swarming non-biting midges and flies^39^,^40^. We therefore assume that the clusters in Fig. 6 represent swarms of these groups over the field. The body:wing proportions of cluster c range between 0.40 and 0.75 and the only insect group caught in the trap that has a body:wing proportions in this range is the group with compact insects. Cluster a and b were both abundant near the trap, and therefore probably positively attracted to it. Cluster c was most abundant in the beginning of the study period, however, the spatial location was not near the trap. A possible explanation for the lack of abundance of species in cluster c near the trap is that this group may not be much attracted to or possibly even negatively repelled by the light trap, consistent with the relatively small abundance of the compact group in the light trap catches (Table 1).

We found pronounced peaks in the general insect activity near the light trap from 22:18–23:18 and 23:18–00:18 local summer time, and an additional peak across the whole measurement time where the light trap was located (Fig. 2).

**Figure 8:**
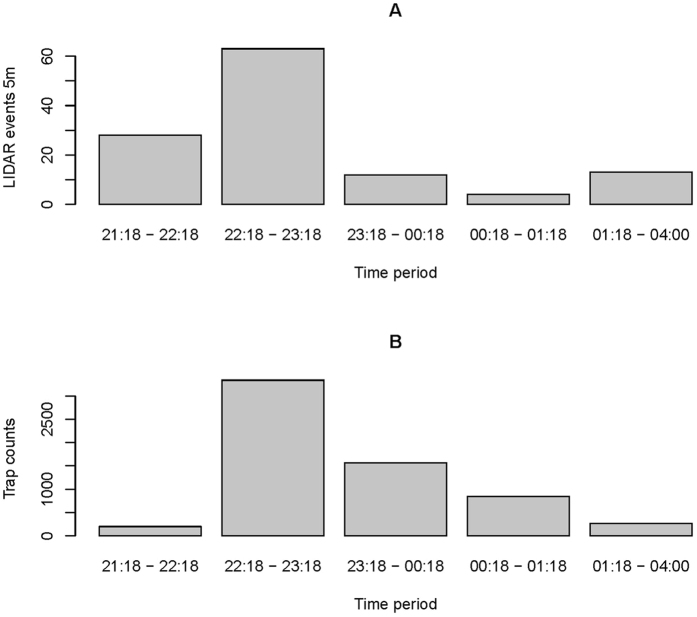
Barplot of (**A**) insect events registered by the lidar up to a 5 m distance from the position of the light trap in five different time periods, and (**B**) the number of insects caught in the light trap during the five time periods. The lidar data were filtered to only show insects with wing sizes greater than 1.5 mm^2^; similar to the insects caught in the light trap.

In Fig. 8 we compare the number of insects caught in the light trap with the number of events recorded in the lidar system within 5 m of the trap. The relative abundance patterns between the methods are similar, but the number of insects caught in the light trap is consistently larger within this area.

We further compared the morphology of insects in the lidar data collected within 5 m range of the light trap with the total insect counts of the light trap for each of the time periods. The smallest wing size measured in the light trap data was 1.9 mm^2^ and belonged to the group Psychodidae (Table 1). We therefore chose to exclude lidar events recorded with wing size less than 1.5 mm^2^ in this comparison to include insects with slightly smaller wing sizes because the morphological measurements were based on small sample sizes.

## Discussion

### Lidar system

The lidar system used in this study proved useful for identifying details of near-surface distributions of small insects. We detected a substantial overlap between the clusters using the body:wing ratio (Fig. 7). This overlap is partly the result of the many directions that insects can enter the lidar beam. When observing an insect from a dorsal, ventral or sagittal view, the wing and body size looks larger than, for example, when observed from an anterior or posterior angle.

### Spatio-temporal distribution of insects

For both the small and fast flapping (e.g. midges, Ceratopogonidae and gnats, Sciaridae), and the larger and slow flapping insects (e.g. moths, Lepidoptera), the activity dropped just before midnight. However, the timing of these events differed (Fig. 3). Small-sized insects showed the greatest activity before and larger insects after 22:00. This activity pattern may be caused by predation of birds, forcing larger insects to wait with their activity until diurnal and insectivorous birds cease their activity during the night. We speculate that this pressure does not apply to smaller insects that suffer less predation from birds.

Estimates of the range of attraction of the Onderstepoort light trap differ considerably, from 2–4 m^41^ and 30 m^42^. In Fig. 2B,C we see an increase in the events recorded towards the light trap position, which we interpret as the light trap attracting insects from a large spatial area. The insect abundance is indeed notably higher up to about 40 m to each side, which provides support for the larger figure and indeed suggests it might be an underestimate. However, even though the insect activity increased in the vicinity of the light trap, this should not be seen as evidence that all nearby insects will enter the trap. Nearby insects could, e.g. be simply attracted to other insects near the trap or prefer to stay at a certain distance from the trap to remain within a specific brightness level. Additional estimates using the lidar system in different environmental settings will be useful to further test this finding, and to validate it.

### Morphological classification of insect groups

The insect composition varied greatly between time periods, showing clear changes in each of the groups´ activities (Table 1). In Fig. 4 we see the measured groups and how they overlap in their body:wing proportions. Given this overlap, it becomes clear that this measure alone cannot be used as a unique identification marker, and thus that a species recognition algorithm has to be included to provide additional more information, for example, the harmonic overtones or the wing beat frequency^19^. Because these parameters were not determined for each group in the current study, it was not possible to separate the groups through a combination of these parameters. Future work should, however, seek to include these additional parameters to investigate the utility of these measures for improved identification of species into distinct groupings.

### Method comparison

Light traps have long been used as a tool to measure the abundance and diversity of flying insects. We here found fairly similar temporal abundance patterns when comparing the light trap catches with the lidar records (Fig. 8). The cost of light traps compared to a lidar system is minimal (200 vs. several thousand dollars for a light trap and lidar system, respectively). The handling time required to count, identify and measure species from light traps can be considerable, however, whereas the lidar system requires specialized personnel to operate it. An advantage of traps is that specimens can be identified with 100% certainty, stored and examined thoroughly, e.g. for inclusion of additional future morphological characters and genetic analyses. A disadvantage of light traps is, however, the requirement for the target species that show positive phototaxis to light traps, which may not prove ideal for all insects. In comparison, lidar data can be analysed using custom made algorithms, which minimises the measurement time needed to estimate morphological characters, as well as minimises observer error and bias (e.g. different people identifying, measuring and counting insects from light traps). Moreover, because the lidar beam does not affect insect behaviour, this method also provides an opportunity to investigate aspects of undisturbed insect movement behaviour, such as their movement towards pheromones^16^, other insects and light signals. However, the lidar system would benefit from some improvements, such as a setup allowing for the continuous scanning across an area without being terminated in a specific place. A possible bias that affect lidar applicability is the influence of the specular reflectance of the optical cross section (which is calibrated using a scatter cross section for a Lambertian diffuse white target), which contributes to the signal. As a result of this, the specular contributions from glittering wings may unfortunately result in a larger optical cross section than the geometrical projection of a diffuse white target. In this study, we reported on the peak-to-peak interval of the oscillatory part of insect echoes, but diffuse and specular contribution could be distinguished by separating lower and higher harmonics^21^ or by implementing co- and de-polarized lidar. Another possible potential bias of lidar is that some insects could potentially fly through the beam more than once, requiring appropriate interpretation of the count data.

In conclusion, here we have shown that the lidar system is a promising new tool for recognizing different groups of small flying insects, and therefore capable of detecting, monitoring, counting and measuring the aerial insect biodiversity and activity, even at night. Our work presents also a first step towards a more advanced use of the lidar system in field of entomology. For instance, several lidar systems could be employed in an integrated way on a country-wide scale to provide an automated insect observation network, to e.g. estimate phenological changes in insect distributions. Tools like this are needed to better understand movement dynamics of flying insects and their associated ecology and biology.

## Additional Information

**How to cite this article**: Kirkeby, C. *et al.* Observations of movement dynamics of flying insects using high resolution lidar. *Sci. Rep.* **6**, 29083; doi: 10.1038/srep29083 (2016).

## Figures and Tables

**Table 1:** Insects categories caught with the light trap.

Records		Time period							Total abundance	Optical cross section (OCS) size (elliptic approximation)		
Group	Common name	21.18–22.18	22.18–23.18	23.18–23.48	23.48–00.18	00.18–00.48	00.48–01.18	01.18–04:00		Wing area (mm^2^)	Body Area (mm^2^)	Mean Body Proportion OCS/(Body OCS + wing OCS)
*Culicidae*	Mosquitoes	4	0	1	1	1	0	1	8	8.53	3.33	0.28
*Psychodidae*	Moth flies	8	40	1	2	6	0	0	57	1.90	0.64	0.25
*Chironomidae*	Nonbiting midges (small)	112	0	0	253	263	109	205	942	2.60	1.02	0.28
*Sciaridae*	Gnats	6	56	22	21	40	4	13	162	2.20	0.65	0.23
*Other*	Flies in a broad sense	28	2974	41	24	18	8	13	3106	2.28	1.02	0.31
*Compact*	Various compact insects (e.g. flies and small beetles)	7	40	0	0	0	0	1	48	4.41	2.03	0.31
*Trichoptera* (*small*)	Caddisflies – small	31	110	98	13	10	1	3	266	5.67	0.86	0.13
*Trichoptera* (*large*)	Caddisflies – large	4	11	0	1	4	4	1	25	26.19	2.81	0.10
*Lepidoptera* (*large*)	Moths – large	0	0	6	0	0	0	0	6	94.98	15.08	0.14
*Chironomidae*	Nonbiting midges (large)	0	102	1043	38	263	104	10	1560	6.78	2.31	0.25
*Aphididae*	Aphids	0	0	1	1	0	0	0	2	5.01	0.92	0.16
*Lepidoptera*	Moths	0	3	0	0	6	5	12	26	119.12	9.00	0.07
*Heteroptera*	Typical bugs	0	0	0	0	0	0	3	3	3.97	1.09	0.21

For each category, the wing area, body area and their proportions are shown. Wings were measured when placed flat on a surface, approximating the wing area as an ellipse based on the longest and widest cross-sectional distance. Bodies were measured from the lateral side, and the area estimated in the same way as the wings.
